# University STEM Students’ Perceived Challenges During the COVID-19 Pandemic

**DOI:** 10.3390/bs15010032

**Published:** 2025-01-01

**Authors:** Yi Ding, Qian Wang, Yingying Yao, Yuwei Liu, Jiayi Wang, Qiong Yu, Emily Marrinan

**Affiliations:** 1 Graduate School of Education, Fordham University, New York, NY 10023, USA; yyao88@fordham.edu (Y.Y.); yliu750@fordham.edu (Y.L.); elm8@fordham.edu (E.M.); 2 School of Engineering, Manhattan University, Riverdale, NY 10471, USA; qian.wang@manhattan.edu; 3 Department of Counseling and Educational Psychology, New Mexico State University, Las Cruces, NM 88003, USA; jwang22@nmsu.edu; 4 School of Education, Queens College, City University of New York, Flushing, NY 11367, USA; qiong.yu@qc.cuny.edu

**Keywords:** university STEM students, perceived challenges, COVID-19 pandemic

## Abstract

University students faced unprecedented challenges and stressors during the COVID-19 pandemic. STEM students experienced unique challenges because many had traditional lectures and hands-on learning in laboratories or field settings. This qualitative study aimed to explore STEM university students’ experiences and difficulties perceived during the pandemic. Secondary analyses of 2 waves of interviews among 12 participants yielded 12 themes across 4 categories that captured the essence of the STEM university student’s experiences: academic and practice, emotional influences, discrimination, and finance. Implications, limitations, and future directions are discussed.

## 1. Introduction

The COVID-19 pandemic has brought significant changes to the climate and format of the educational system. In 2020, the percentage of undergraduate students in the United States who took distance education courses exclusively was 44% in 2020 in comparison to 15% in 2019 (National Center for Education Statistics, 2023). While online education has provided many opportunities for students who are willing to pursue a higher academic degree in this special period of time, it also raises a myriad of challenges. Other than potentially low accessibility due to a lack of available digital devices and poor technological skills, online education also requires access to a private and quiet place to ensure students can concentrate on their learning (Aisha & Ratra, 2022; Rakes & Dunn, 2010). For students who attend in-person academic programs, the absence of the physical presence of facilitators and instructors during an emergency situation can limit social interaction or create a sense of isolation, which, consequently, can lead to inactivity, low motivation, and decreased self-regulation among students (Rakes & Dunn, 2010). Although American employers’ perceptions about online degrees have shifted over time, a wide range of opinions on online learning remain and some employers still hesitate to hire or consider an online degree holder (Friedman & Claybourn, 2022). The pandemic also caused socioeconomic issues among students, especially those from disadvantaged communities (Fernandes, 2020). It is worth noting that ethnic minority students were disproportionately influenced by academic-, financial-, and COVID-related stressors (Reyes-Portillo et al., 2022).

Although plenty of research addressed these issues among college students, Master’s students, and doctoral students, in general, research focusing on students majoring in STEM remained limited even though they are very likely to encounter more difficulties regarding the nature of their major. Kalkbrenner and Miceli summarized a few barriers for STEM students to seek mental health support (Kalkbrenner & Miceli, 2022). First, STEM students are less likely to self-recognize signs of mental distress and they access mental health services at lower rates than their peers. Second, STEM majors are challengining and competitive by nature. Third, some STEM students might exhibit higher levels of maladaptive perfectionism, which can lead to higher levels of mental distress. Therefore, this paper will review these unique challenges faced by STEM students during the COVID-19 pandemic and conduct a theme study focusing on examining these challenges using qualitative methods and analyses. Multicultural concerns regarding unique challenges for underrepresented minority STEM students will also be discussed. By understanding more about these difficulties faced by this specific group, relevant organizations will be able to help these students in a way that can better accommodate their actual needs, especially for those universities that are still offering online or other asynchronous classes.

### 1.1. Particular Challenges of STEM Students During the COVID-19 Pandemic

#### 1.1.1. Lack of Hands-On Learning Opportunities

Unlike non-STEM majors, STEM education encompasses a lot of subjects that require lab or field experiences. While lectures provide students with theories and concepts, practice in labs and fields can improve STEM students’ practical skills and help them have a deeper understanding of the lecture content. Therefore, hands-on lab and field practice are indispensable to STEM education. However, on-campus laboratories were unavailable due to the campus closure during the pandemic. This lack of these hands-on opportunities had a negative impact on STEM students.

Most schools adopted virtual laboratory courses as substitutes for traditional on-campus laboratory classes. However, many STEM students did not perceive them, as an alternative, as effective as on-campus ones. A study showed that 51.2% of the participants (*n* = 41) had a highly negative experience in their virtual laboratory classes. Out of the respondents who believed that the quality of their instruction was reduced, 37.5% (*n* = 8) attributed it to the absence of hands-on learning opportunities. The extent to which students enjoyed online experiences was negatively correlated with the lack of field experiences (*r* = −0.34, *p* < 0.05). This result indicates that as the participants’ liking for online classes decreased, they perceived a greater absence of field experiences (Desrochers et al., 2020). Hou et al. collected data on their perceptions of online laboratory classes from 644 STEM students at California State University, San Bernardino (Hou et al., 2021). In this study, 30.95% of students felt extremely or somewhat dissatisfied with their online laboratory courses, and more than 50% of the respondents reported a significant reduction in learning efficiency in online laboratory classes. In addition, “laboratory resources” were identified as one of the top five factors that affected their study during the pandemic. From an interpersonal perspective, the lack of hands-on learning experience also minimizes opportunities for students to collaborate with their peers on the fieldwork, as 67% of a random national sample of 1008 undergraduates rated their teamwork experience as worsening due to the online setting (Means & Neisler, 2020).

#### 1.1.2. Delay in Graduation

Many STEM students reported delaying graduation due to the pandemic. In a study of 4603 U.S. STEM students, about 7.6% of the undergraduate participants, 18.0% of the Master’s participants, and 35.5% of STEM doctoral students postponed their graduation due to the COVID-19 outbreak. The leading institutional factors behind the graduation delay were identified as (a) limited access to academic facilities or resources and (b) delayed coursework or degree-required projects, which were reported by 61.9% and 40.8% of the respondents who delayed their graduation, respectively. While the study did not explain how access was limited and how the coursework was delayed, it is possible that the absence of laboratory and field resources contributed to the two identified factors for delayed graduation (Saw et al., 2020). Forakis et al. investigated STEM undergraduates at the University of Alabama at Birmingham who were enrolled in a general chemistry laboratory course and found that nearly 20% of the participants intended to suspend their coursework, which would lead to a delay in graduation (Forakis et al., 2020). The research also reported that 12% of the participants missed extra-curricular chances including off-line summer research internships and shadowing programs, which significantly contribute to retention among STEM students.

On the other hand, through previous published work, researchers pointed out that academic performance was significantly higher for STEM students who attended in-person classes than those who attended online classes (Espinoza, 2022). To test this statement, professors from the University of California Irvine use a common final exam (CFE) in an Introduction to Biological Introduction Sciences course to assess the student’s understanding level of the subject. They found that students who attended in-person courses had higher passing rates and higher average scores on the exam, which aligned with the findings in previous research. Without being able to achieve a passing grade in a class, students generally need to take that course again, which will likely cause a delay in graduation. As discussed above, there is no doubt that taking online courses during the pandemic increased the risk of delay in graduation for STEM students. Furthermore, academic distress is a main predictor of lower enrollment and completion rates in STEM fields (Daker et al., 2021). Muenks et al. pointed out that this academic distress will simultaneously contribute to STEM students’ psychological distress (Muenks et al., 2020). And, vice versa, STEM students’ rising psychological vulnerability predicted a further decrease in class attendance, higher dropout intentions, and less class engagement. The next section will discuss more detailed information about STEM students’ psychological distress.

#### 1.1.3. Mental Health Concerns

While mental health concerns are common among students from all majors during the pandemic, the nature of STEM courses aggravated students’ mental health issues. The high level of expectations, stress, and rigor of STEM courses can induce or exacerbate mental health issues (Lipson et al., 2016). Furthermore, students enrolling in STEM majors tend not to notice warning signs of their psychological distress (e.g., lack of motivation, mood changes, lack of concentration, withdrawal, and sleep or appetite changes) and they are less likely to access mental health support services in comparison to their non-STEM peers (Kalkbrenner & Miceli, 2022). However, further research focusing on this issue is needed, as there is a dearth of literature on why STEM students are prone to not using counseling services. While STEM students lack the motivation to seek professional help, Rice et al. also reported that STEM students are prone to have mental distress correlating to their higher levels of maladaptive perfectionism (Rice et al., 2015). One survey conducted by the University of California, Berkeley showed that more than 40% of graduate students in STEM are depressed compared to 29% of all graduate students. At the same time, the COVID-19 pandemic worsened the situation by increasing the ratio of STEM students with depression and anxiety symptoms to 50% (as cited in Li & Wu, 2021).

One possible explanation of this phenomenon is that mathematics is the foundation for all other STEM fields. A lack of competitive mathematical skills may affect STEM students’ performance in other STEM subjects and their future career development (Maass et al., 2019). While math anxiety is one of the specific challenges faced by some STEM majors, it was worsened during the pandemic. Math anxiety first appeared as “mathemaphobia” in the 1950s to describe the fear and dislike for math (Gough, 1954). Then, in the 1970s, Richardson and Suinn proposed the concept of “math anxiety” and defined it as “a feeling of tension and anxiety that interferes with the manipulation of numbers and the solving of mathematical problems in a wide variety of ordinary life and academic situations” (Richardson & Suinn, 1972, p. 551). A study of 53 STEM undergraduates enrolled in Calculus classes during the 2020 summer semester found that math anxiety was prevalent among mathematics students during that time. To be specific, about 20% of the participants reported having math anxiety during that semester. Additionally, freshman and sophomore participants expressed greater levels of math anxiety compared to their junior and senior peers, with respective average rates of 27.4% and 15.5% (Soysal et al., 2022). Mendoza et al. also found an increase in the level of math anxiety among mathematics students at the Universidad Nacional de Chimborazo, which may be associated with a statistically significant decrease in the understanding of class contents taught virtually (Mendoza et al., 2021).

Another explanation is that STEM students’ positive attitudes toward science decreased more during online courses. One pretest–posttest study assessed 73 STEM undergraduate students’ attitudes toward science via the Colorado Learning Attitudes about Science (CLASS) before the start of online courses and at the end of the 2020 spring semester. The results showed that participants’ positive attitudes toward science dropped significantly at the end of the 2020 spring semester. To be specific, compared with the pre-survey, 87.6% of the participants exhibited a decrease in their favorable attitudes toward science scores, and 60% of the participants’ negative attitudes toward science scores increased in the post-survey. Regarding the population of all students, a meta-analytic result based on forty published papers (*n* = 98,558) showed that only 13.3% of students held negative attitudes toward online courses during the pandemic (Liu et al., 2022).

#### 1.1.4. Unique Challenges for Underrepresented Minority STEM Students

Female students, students from minority backgrounds, first-generation college students (FGCS), and students with low socio-economic status (SES) backgrounds encountered more obstacles than their STEM peers during the pandemic. Kahn et al. investigated 186 STEM students at a university in the northeast region of the United States in the second half of the 2020 spring semester (Kahn et al., 2022). The study showed that the transition to online STEM education had a disproportionately negative impact on students from underrepresented groups. Particularly, Black students from low SES backgrounds faced the greatest challenges with reliable internet access, a lack of study space, a higher likelihood of losing contact with their teaching aids, and increased caregiving responsibilities, which hinder their ability to focus on schoolwork, especially for these second-generation female immigrants. Each of these factors could potentially contribute to students’ decreasing self-efficacy in STEM subjects during the pandemic. Saw et al. found that Hispanic and Black STEM undergraduates are less likely to graduate on time due to the pandemic compared with their White and Asian peers (Saw et al., 2020). Specifically, while the percentage of White and Asian students who delayed their graduation was 6.0% and 6.3%, respectively, 12.7% of Hispanic and 10.3% of Black participants postponed their graduation.

On the other hand, although Asian students were still prone to graduate on time, it did not indicate that they were encountering fewer challenges than other ethnic minority groups. According to the Federal Bureau of Investigation (FBI), hate crimes against Asian people residing in the United States increased 77% from 2019 to 2020 (Findling et al., 2022). In addition to the direct harm caused by those crimes, living in an environment that became unwelcome and less secure was also inducing extra stress among Asian students. Nationwide, the prevalence of depression symptoms in the U.S. was higher in every demographic category during COVID-19 than before COVID-19 (Ettman et al., 2020). Mild depression symptoms prevalence was 16.2% before COVID-19 compared with 24.6% during COVID-19, reflecting a 51.85% increase (Ettman et al., 2020). For the Asian population, Lozano et al. found that the prevalence of depression symptoms increased from 9% before the pandemic to 21% during the pandemic among two Asian American ethnic groups, reflecting a 133.33% increase, and discrimination was one of the main contributing factors (Lozano et al., 2022). The question of whether the dramatically increased psychological distress stemming from anti-Asian racism has extended to other ethnic groups remained unanswered.

## 2. Method

### 2.1. Participants

The 12 participants (6 females and 6 males) in this research were selected from a larger sample of 36 students studying in multiple universities in New York City through convenience sampling via professional networks of professors and students. The original large-scale study recruited 36 participants for interviews (e.g., Asian American students, international students, STEM students, and some other students) and only 12 of them were STEM students. The 12 participants were in the age range from 19 to 25 years old; 7 of them were Asian, 4 were white, and 1 was biracial. The participant recruitment process was largely based on the researchers’ professional networks. The researchers were mostly Asian or Asian American, which led to more Asian or Asian American participants as an unintended outcome.

### 2.2. Procedure

The original larger-scale study recruited potential participants through an online Qualtrics survey. After providing consent and completing the self-report survey, respondents who were interested in participating in follow-up interviews could provide their contact information and availability at the end of the survey and were randomly selected by a random number generator. Interviews were conducted semi-structured and open-ended, and elaboration of participants’ responses was elicited. A USD 10 Amazon gift card was given to the participants as compensation after each interview. The interviews were conducted in April 2020. The average length of the audio-recorded Zoom interview lengths ranged from 16 to 26 min (M = 20.63, SD = 5.38).

### 2.3. Materials and Analysis Approaches

The primary interview inquiries were as follows: (1) Could you share the major difficulties you encountered during the COVID-19 pandemic? (2) Have you encountered any COVID-19-related discrimination, hate crimes, unpleasant experiences, or anything else? (3) What would you like to see in terms of support at the community level? (4) What would you like to see in terms of support at your university and program levels? We used Moustakas’s transcendental phenomenology method to describe university STEM students’ experiences by highlighting significant statements (horizontalization) (Moustakas, 1994). A systematic interpretation of interview transcripts and extraction of common themes across the interviews developed clusters of meaning from the 12 participants’ interviews about their experiences as university STEM students during the COVID-19 pandemic. This approach often utilizes epoche or bracketing, and it allows the intrinsic meanings to emerge within their own identities. This qualitative method explored the participants’ unique lived experiences and co-construction was utilized to interpret findings in order to gain a clear sense of the data. The primary researcher bracketed her own experiences in order to engage from a fresh perspective toward the phenomenon of interest of the current study. A secondary researcher reviewed the interpretations for validation purposes.

## 3. Results

**Figure 1:**
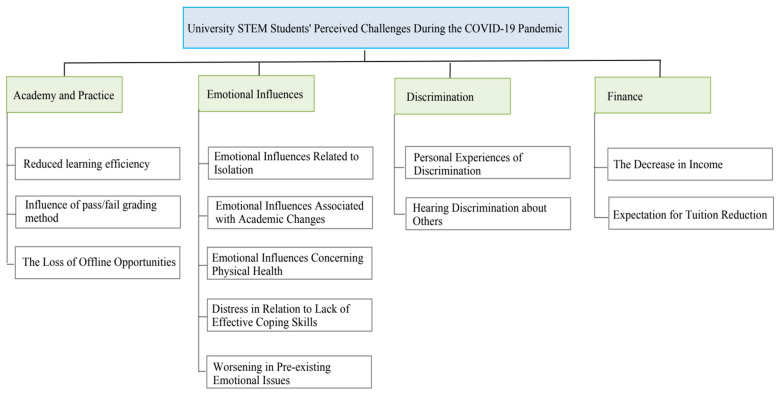
STEM students’ perceptions model.

A model illustrating STEM students’ perceptions of the first several months of the pandemic emerges (see Figure 1). The first category, “Influences on Academy During the Pandemic”, and its three themes explore how the start of the pandemic affects participants’ learning efficiency, grading, and offline research or practical activities. The second category, “Emotional Influences”, and its five themes explore how the pandemic negatively affected students’ emotions. The third category, “Experience of Discrimination”, and its two themes explore whether the participants confront discrimination and how the experience of discrimination influences them. The final category, “Financial Influence”, and its two themes explore the pandemic’s effect on participants’ financial status.

### 3.1. Influences on Academy and Practice During the Pandemic

#### 3.1.1. Theme 1: Reduced Learning Efficiency

During the pandemic, schools offered their courses online. Many students did not adapt well to the virtual courses, leading to a reduction in learning efficiency. Some students find it hard to focus after the change in study environment. YN said that “My student difficulty is, just, hard to concentrate at home”. AN shared a similar experience as YN: “I haven’t really been able to concentrate well on listening to lectures or reading essays I’m supposed to be analyzing, so I feel like my concentration has taken a hit”. RS shared her experience of being distracted at home:

I do notice that I have been a lot less productive than I was on campus…Doing it at home, especially since I share a room with my sister, it’s somewhat difficult to get privacy sometimes in that sort of environment.

A potential reduction in interaction among students and helpful resources in virtual teaching also influenced students’ study efficacy. AN stated the following:

I feel like I learn best by talking it out, interacting, and asking the TAs questions, but ever since I had to go back home, I have really been limited in my ability to interact with my fellow classmates. I’ve been doing zoom meetings and facetiming and other video chatting platforms, but it’s really not the same. I feel like I’m not getting the same level of interaction, and therefore not really delving super deep into the material that I’m supposed to be learning.

For XL, she felt other students’ decreasing engagement through decreased frequency in asking questions and efficiency in teamwork tasks due to the change in environment: “Students usually ask questions often in the physical room, but instead of so, we are using virtual classroom, so there are not many students asking questions, so like interact with professors”. She also stated that teamwork through online apps “might not be that efficient compared with physical meetings”.

In addition, learning efficiency also decreased by the reduction in teaching quality. Some professors did not make a good transition with virtual courses, influencing students’ experience. ZW stated that “The transition to online learning has been the biggest thing that’s been stressful because every professor has been handling it a little differently—some are great with this, some aren’t”. Some professors even replace virtual live courses with recorded videos. SH shared that “Some of my professors just upload their teaching video to the CANVAS. And I don’t think that’s as good as the online lectures”.

Virtual classes also influenced students in some other aspects. ZW stated that “The grading situation has been changed—which projects are delayed, hard to know when is stuff due”. She also added that her experience was worsened due to a technical error.

Many people have inadequate study time during the pandemic due to their changing life patterns. XL said the following:

I usually ask for delivery food. After the coronavirus, there are not many restaurants opening for delivery, they are all closed. So…I might be facing the issue with surviving, like food. We have to go grocery shopping and buy food and cook for ourselves. It’s kinda wasting time.

For CW, more time and energy was spent on taking care of her mother, who was immunocompromised:

So, I definitely found the transition to online education really difficult because when I came back here I suddenly had a whole family to look out for too and I couldn’t be as individualized—my mom is more immunocompromised so I would be the one who had to go out for groceries and things like that.

#### 3.1.2. Theme 2: Influence of Pass/Fail Grading Method

During the pandemic, many colleges change their grading method to the pass/fail form in response to virtual courses. Students express their different feelings and attitudes toward this change. CW, who planned to apply to medical schools after graduating from college, expressed her worry as some of the medical schools she wanted to go to might not accept pass/fail grading methods, which would be very likely to disrupt her academic route. Simultaneously, if she did not select the pass-or-fail grading method, it would increase her risk of receiving lower grades, which put her in a dilemma. CW said the following:

The pass/fail option, I understand that the university was very accommodating with allowing us to make classes pass/fail, but I think they also put us in an uncomfortable position of making an uninformed decision given that we don’t have our final grades yet, so some of us are taking a big gamble…If you are pre-med or pre-law or pre-grad, they are much more ready to accept pass/fail grades if it was mandated by the school, whereas if they know that you elected to take the classes to pass/fail they may take a student with a letter grade over you.

Admittedly, the pass/fail grading method might have certain advantages to some extent. Opposed to CW, some students held positive attitudes toward this grading change. RS said the following: “Also everything is pass/fail. That has been really helpful, especially for lab classes”. Kan, a business student, shared a similar positive attitude toward the pass/fail grading:

They’ve offered pass/fail—I don’t know if it was all of [University] but it was the business school, so that was good considering everything has been changed. I would’ve liked to see all the schools do that, I don’t know if they did, but that was something good they could have done.

#### 3.1.3. The Loss of Offline Opportunities

Most offline research and practical opportunities stopped during the first several months of the pandemic, affecting a lot of students. Some, like Kan, lost their internship. He said the following: “The work that I was talking of, I guess you could say it’s an internship, which was disrupted”. RS shared a similar experience: “I also had a summer internship canceled, which was a little bit unfortunate”. The loss of offline opportunities affected seniors more. LM, a senior in the process of applying for graduate school, stated the following:

Another difficulty is that I’m a senior…I had an internship at NYC Mount Sinai and I was furloughed—I don’t know if I’ll get the internship back once this is all over—I presume not. So that’s been stressful. I’m in the process of applying to graduate school, so the internship being something that I can’t use as experience is frustrating.

For doctoral student DN, the loss of practicum may affect his graduation. He said the following:

You made a point about practicum and that social relevance to me. In my program, that practicum piece is a big part now. So, it’s kind of thinking of ways to be creative about this and for it not to affect us in any more aggressive way than it possibly already is, and the potential delaying graduation, but of course, these are the things that have been outside… mostly outside of our control.

Not only did students lose internships, but it was also not easy to find suitable research opportunities, which are critical to STEM students. CW expressed the willingness of her school to provide more online-friendly research opportunities and clarifications regarding the next step:

If they would be able to provide us with information on where to look to find online-friendly kind of like clutch times for getting all of your research in, getting your experiences in…and since it’s so unclear about whether or not we will be online next semester, or what’s going to happen with research that’s online now, or like during the summer time is it going to be online, just kind of like offering more clarification of like, “hey guys this is what is going to be happening”, or “this is what we still have the resources to help you with your project in an online format”, or “here are the professors who are willing to work with it online”, things like that. That would be helpful…For the neuroscience program…there is a mandatory research requirement.

### 3.2. Emotional Influences

#### 3.2.1. Theme 1: Emotional Influences Related to Isolation

The most frequently reported emotional problems during the pandemic were related to isolation. YN felt lonely when isolated at home: “My major challenges…probably staying at home too long. …probably some social activity is much lesser than before. I mean some lonely situation”. SH had the same feeling as YN: “I just feel…the feeling of loneliness at home, cuz I don’t have a roommate, so…just…I’ve been staying in my apartment for over a month, yeah, not talking to any people, I mean like face to face. That’s kind of challenging”.

AN also felt depressed being isolated at home:

Socially, I have been basically just at home for over a month now. I haven’t been able to see any of my friends in person since coming back home. I think maybe my emotional health has taken a bit of a toll just because I’m someone who really depends on these person-to-person interactions and I really haven’t been getting much of those ever since the pandemic started.

For CW, she missed her friends during the isolation:

I went from living in one of the most social cities in the nation to going back to my isolated house on Long Island where I am about a twenty-minute drive away from any type of social interaction with the general public…It was difficult because in the beginning stages of them asking us to leave, it was initially just like “oh you’ll be back in a couple of weeks, so just take stuff to be away for a couple of weeks and then we will be back”. so we weren’t totally sure about when we would see our friends again after the situation started to develop because the university sent out emails saying that it might be pushed a little farther back and now you might be away for the rest of the semester.

#### 3.2.2. Theme 2: Emotional Influences Associated with Academic Changes

Virtual courses affected students’ learning efficiency and influenced their moods. ZW felt stressed during the transition. He stated that “The transition to online learning has been the biggest thing that’s been stressful because every professor has been handling it a little differently- some are great with this, some are not”. As a senior student, LM had a lot of negative emotions about the loss of the senior week and her internship:

I’m a senior, so moving to online classes and not getting a graduation or senior week has caused a lot of stress, sadness, frustration, and anger…I’m in the process of applying to graduate school, so the internship being something that I can’t use as experience is frustrating.

#### 3.2.3. Theme 3: Emotional Influences Concerning Physical Health

The start of the COVID-19 pandemic elicited some students’ worries about health. XW said she had “stress and concerns about my health”. RS expressed her concern about her grandmother’s health: “I do have a grandmother, so it’s been a little bit worrying, seeing how she’s been doing. She’s been doing alright. It’s just that she is older and she does have some health conditions”.

#### 3.2.4. Theme 4: Distress in Relation to Lack of Effective Coping Skills

Many students expressed their fear and worries regarding discrimination and hate crimes but do not have effective coping skills to ease their negative feelings or are not positive that they can make a difference in their current situation. For example, XW chose to stay at home as much as possible, and DN said the following:

I think. I mean… there’s no… well, I wanna say there’s not so much you can do. There are something you can do, but to do something actively, you know… I wouldn’t allow discrimination to um… occur in the presence of myself, but I mean, part from that is kind of limited, or limited in how you can really… I don’t know… like… have an active role and address it.

On the other hand, some students have more effective coping skills but do not feel they have encountered many major challenges. For example, PS said the following:

I am trying to call them mostly video call them. I am engaging in online courses learning more things. I am getting more time to do cooking for myself. That is it I am talking to people and connecting with them and building some of my networking skills.

#### 3.2.5. Theme 5: Worsening in Pre-Existing Emotional Issues

The pandemic exacerbated the pre-existing emotional problems of students. LM stated: Mostly I had pre-existing mental health issues and they kind of got exacerbated during all of this so it’s been challenging to keep myself positive and optimistic. So that would be the biggest thing.

### 3.3. Discrimination

#### 3.3.1. Theme 1: Personal Experiences of Discrimination

At the beginning of the pandemic, some Asian students confronted direct or indirect discrimination. RS stated the following:

…when I was still on campus, my school is in a pretty large city. So walking to a grocery store that was somewhat nearby. I remember there was a group of, I think middle or high school students, I’m not entirely sure—they would say mean things to, especially, Asian people walking around, I noticed…I think it was just a group of maybe five or six kids basically. They basically acted all dramatic and scary, like “Oh my gosh, they have coronavirus!” and they would run around or run away or whatever. It made me really uncomfortable.

AN felt that she experienced indirect, nonverbal discrimination. She said the following:

Nobody has said anything verbally or done anything physically, thank goodness. I find that whenever I do go out to grocery shopping sometimes I get strange looks, kind of cold looks. The air that other people give off is really not friendly.

#### 3.3.2. Theme 2: Hearing Discrimination About Others

Though not experiencing discrimination themselves, some students knew that their friends experienced discrimination. ZW stated the following:

I’ve heard countless stories from a couple of friends of mine who are East Asian—I don’t think any of them specifically faced overt discrimination, but I remember at least one of them their mother wanted them to come home or go away from the city—they go to college in the city—and their mother was just like ‘can you come home to—’ I don’t know where they’re from but yeah, their mother wanted them to come home because they were afraid they would face discrimination in the city and I’m sure that concern is very legitimate.

CW shared the following:

I knew a friend who is Asian, and she lives in Colorado, not in New York or anything where people might be more educated or whatever. So she was like living in the more rural area of Colorado, and she’s walking down the street with her family and her neighbors pointed at her and said “oh look there’s four of them” and something like that.

### 3.4. Finance

#### 3.4.1. Theme 1: The Decrease in Income

Some students reported a decrease in income as a result of the pandemic. DN regarded the loss in income as his major difficulty during the pandemic. CW also reported the decrease in income and its influence on her financial burdens:

Starting with student employment, first, I know a lot of students supported themselves financially through university jobs, including me, but once we were sent home, those positions we couldn’t really continue with. They did, I think, say that if you are federally employed by the university then you could continue to get paid, but there’s a difference between an institutional job and a federal job at the university, and I have an institutional job, so I don’t know if I’m going to continue to getting paid. That was a dominant source of income for me to take care of grocery expenses or to help with loan payments, school supplies, things like that. So if there is something in place for those who have an institutional job, that would be great.

#### 3.4.2. Theme 2: Expectation for Tuition Reduction

Several students mentioned that they wanted tuition reductions because of the change to online courses. XW felt that students should receive refunds in tuition because they could not access campus resources: “I know everyone is having a tough time right now, but I would be glad if the university is going to…give some kind of refund in respect to the tuition, cuz we are not using any resources on campus”. SH hoped for a return in tuition fees because of the online teaching quality: “I hope my university can return some college fee, tuition fee. Everything is changed online, and I think the tuition quality is not as good as before…the teaching quality…online teaching quality”. DN also wanted a reduction in tuition fees because of the incomplete experiences of online courses compared to courses on campus:

…For my school at least, the summer has been confirmed to be online. Um… and so, for me, I am wondering: “well, you know… what does this mean in terms of the tuition that’s being that’s being expected to be paid now”. …you know, what does that mean for tuition? I mean mainly it’s gonna be online because we are losing an important aspect of the program that I think is reflective in the fees…

## 4. Discussion

### 4.1. Reduced Learning Efficiency

Reduced learning efficiency related to distractions, inadequate learning time, lower teaching quality, and technical errors in remote learning were identified as the most reported concerns among participants, consistent with some recent studies. In a study including 584 STEM students, 33.7% of the participants identified quality of education as a worse aspect of virtual learning in Spring 2020, 10.8% perceived distractions, and 8.2% complained about technology issues (Selco & Habbak, 2021). In research about STEM undergraduates’ learning experiences in Spring 2020, 13% of the respondents felt that instructors were unprepared for class, 13% reported being impacted by technology failures, and 15% complained about pre-recorded lectures. A student reported an instructor using an mp3; another complained that an instructor even used recorded lectures from 2014 (Pagoto et al., 2021).

### 4.2. The Pass/Fail Grading

The change in grading method to the pass/fail option is a policy adopted by many universities in Spring 2020. Our results showed students’ conflicting attitudes toward the pass/fail grading method. Some students held positive attitudes toward pass/fail grading, believing it reduced their pressure, which aligns with some recent studies. In the study conducted by Pagoto et al., STEM undergraduate respondents identified the use of pass/fail grading as a lenient action (Pagoto et al., 2021). However, a student expressed concern about the potential impact of the pass/fail grading on applications to graduate schools, law schools, or medical schools and her difficulties in choosing between numerical grading and pass/fail grading. Her concern was not unreasonable. Although no direct data are available on the impact of pass/fail grading on medical or graduate school applications, research on residency applications suggested that it could have an influence. In 2020, the grading method for the United States Medical Licensing Examination (USMLE) Step 1, which is significant in assessing residency applicants, changed from a numerical score to pass/fail. A survey of 257 staff members in the Department of Otolaryngology revealed that the change might benefit top-ranked school applicants during the residency application and match process but negatively impact international or underrepresented applicants (Goshtasbi et al., 2021). The survey results indicated that pass/fail grading could lead to unfair advantages for students from top-rated programs, as they might be considered more competitive without objective numeric scores. Similarly, pass/fail grading could have comparable impacts on medical school or STEM graduate program applications. To help students who are concerned about the change in grading methods, we recommend career counseling center provide specific services for them.

### 4.3. Emotions and Coping

“Emotional Influences” is a major theme that interconnects with other themes in our study. Specifically, academic changes, such as reduced learning efficiency, the transition to online learning, or the change in the grading method, were associated with stress, frustration, sadness, and anger. Experiencing discrimination personally or hearing others being discriminated against was related to worry, fear, and even scary. Extensive research has demonstrated the impact of academy and discrimination on general college students’ mental well-being. Empirical data revealed that STEM students’ academic well-being during the COVID-19 pandemic was lower than it was before the onset of COVID-19 (Aarntzen et al., 2023). Academic stresses, including a reduction in focus on academic work, difficulties in online learning, and fear of poor academic performance, were related to increased levels of depression, anxiety, general stress, and bodily distress (i.e., somatization) (Fruehwirth et al., 2021; Kecojevic et al., 2020). A study conducted by Teng et al. indicated that nearly 70% of East Asian college respondents feared being harassed or a sense of unsafety due to COVID-19. In addition, the study showed that discrimination fear was related to higher levels of depressive and anxiety symptoms (Teng et al., 2023).

Furthermore, our study points out a relationship between lack of effective coping skills and perception of distress. To help students cope with negative emotions and facilitate successful academic performance, we recommend that the college counseling center offer virtual workshops that provide psychoeducation and psychological support or teach necessary coping skills to help students overcome diverse obstacles. Several skills could be incorporated into the workshop. First, mindfulness could be a good way to both reduce stress and various negative emotions and keep focus. Extensive evidence has demonstrated the effect of mindfulness-based interventions (MBIs) in improving various psychological outcomes, including depression, anxiety, stress, and many other psychological symptoms (Chiesa & Serretti, 2009; Hofmann et al., 2010). A higher frequency of a mindful state has proven to be associated with higher positive affectivity, life satisfaction, self-esteem, and self-actualization (Brown & Ryan, 2003). Additionally, a study conducted by Mesghina et al. indicated that mindfulness could reduce mind wandering related to COVID-19 distress during the lesson (Mesghina et al., 2021). Second, time management skills are important, as several participants in our study reported inadequate learning time due to the additional responsibilities of taking care of family members or increased cooking time resulting from the lack of access to food delivery. Third, communication skills could also be helpful. In our study, some students were unsatisfied with professors’ teaching styles; one student reported being easily distracted by family members. Effective interaction skills could help students communicate their feelings and thoughts with professors or family members, enhancing their experiences.

University professors would benefit from training to enhance the effectiveness of online teaching. In addition, university mental health centers should make university professors more aware of available resources (e.g., university counseling center, mental health support), then professors can provide mental health resources to students when needed.

Groups that focus on psychological support can also be beneficial, as supported by research. Nacif et al. assessed the effect of an online group coaching program among postgraduate students from the University of East London during the pandemic (Babineau, 2018). Participants reported experiencing a safe environment, feeling related to others, a sense of belonging, and improved self-awareness. It is important to create a community where all students can feel safe and valued. In total, 7 out of 12 participants were Asian in this study. Babineau’s review of the literature suggested that underrepresented students persist through college at lower rates than their peers (Nacif et al., 2023). Numerous factors impact their persistence and dropout rates in college, including social and cultural, economic, academic, and situational barriers (Nacif et al., 2023). We specifically recommend providing multi-tiered support for unrepresented students to help them navigate through college. For example, resources and support should be provided at the advisor/faculty level, program and department level, and the university level.

### 4.4. Financial Difficulties

In our study, several students expressed their financial burdens and desires for a tuition reduction. Although no direct data about STEM students’ financial difficulties are available, research has demonstrated general financial burdens among college students during the pandemic. A study including 4714 college students from schools in New York and New Jersey indicated that nearly half of the participants lost their jobs or had their work hours reduced, 37% reported significant negative financial impact, and nearly 30% expressed their concerns about access to food (Reyes-Portillo et al., 2022). Students’ desire to have tuition reductions is reasonable: extensive studies have shown a reduction in the quality of online education. Colleges and governments should find effective ways to help students relieve their financial burdens.

## 5. Limitations and Conclusions

There are several limitations to this study. First, selection bias may exist in relation to the small sample size and a lack of diversity in terms of race/ethnicity, gender, sexuality, graduate/undergraduate, and social class. Further research should enlarge the sample size and diversity to fully comprehend the impact of the intersectionality of these factors on the experiences of STEM students during the pandemic. Second, our research was limited to the New York metropolitan area. Research should be expanded to other areas in the U.S. to get a bigger picture of STEM students’ perceived challenges during the pandemic. Third, we did not compare the experiences of STEM students and non-STEM students in our study, making it hard to learn the experiential differences between these two populations. Finally, many participants provide a lot of details about different kinds of difficulties but do not identify their specific feelings and emotions toward these difficulties. For example, several participants talked about their willingness to a tuition fee reduction but did not express their feelings toward it. This may lead to a lack of comprehensive understanding of the influences of different kinds of obstacles on students’ mental well-being.

This study explored the unique experiences of university STEM students during the COVID-19 pandemic and the impact of their experiences. The results suggested that STEM students encountered experiences such as emotional influences, COVID-19-related discrimination, and financial difficulties similar to other university students’ experiences (Chiesa & Serretti, 2009). In addition, STEM students also encounter unique challenges due to the nature of their majors, such as requirements of lab experiences and field placement, which might not apply to students whose studies do not require such hands-on experiences. Future research should continue to explore perceived challenges and difficulties of STEM students in higher education and develop university-level counseling services or interventions to improve university STEM students’ overall experiences and subjective well-being during unprecedented situations. In addition, support should be provided at the school or program level by enhancing mental health awareness among academic advisors serving STEM students.

## Data Availability

Due to privacy concerns mentioned in the IRB protocol, the data associated with this study cannot be provided to the public without the supervision of the researchers. However, individual researchers who are interested in obtaining access to the data for individual use are encouraged to contact the corresponding author.
